# A Personalized Behavior Learning System for Human-Like Longitudinal Speed Control of Autonomous Vehicles

**DOI:** 10.3390/s19173672

**Published:** 2019-08-23

**Authors:** Chao Lu, Jianwei Gong, Chen Lv, Xin Chen, Dongpu Cao, Yimin Chen

**Affiliations:** 1 School of Mechanical Engineering, Beijing Institute of Technology, Beijing 100081, China; 2 School of Mechanical and Aerospace Engineering and School of Electrical and Electronic Engineering, Nanyang Technological University, Singapore 639798, Singapore; 3 Department of Mechanical and Mechatronics Engineering, University of Waterloo, 200 University Avenue West Waterloo, Waterloo, ON N2L3G1, Canada

**Keywords:** autonomous driving, driving behavior, human-like control, artificial neural network, reinforcement learning

## Abstract

As the main component of an autonomous driving system, the motion planner plays an essential role for safe and efficient driving. However, traditional motion planners cannot make full use of the on-board sensing information and lack the ability to efficiently adapt to different driving scenes and behaviors of different drivers. To overcome this limitation, a personalized behavior learning system (PBLS) is proposed in this paper to improve the performance of the traditional motion planner. This system is based on the neural reinforcement learning (NRL) technique, which can learn from human drivers online based on the on-board sensing information and realize human-like longitudinal speed control (LSC) through the learning from demonstration (LFD) paradigm. Under the LFD framework, the desired speed of human drivers can be learned by PBLS and converted to the low-level control commands by a proportion integration differentiation (PID) controller. Experiments using driving simulator and real driving data show that PBLS can adapt to different drivers by reproducing their driving behaviors for LSC in different scenes. Moreover, through a comparative experiment with the traditional adaptive cruise control (ACC) system, the proposed PBLS demonstrates a superior performance in maintaining driving comfort and smoothness.

## 1. Introduction

During the last several decades, considerable efforts have been made to design and develop highly autonomous vehicles that can drive with little or even no interventions from human drivers. However, the overall architecture for designing autonomous vehicles has not been improved too much. Most of the existing autonomous vehicles share the same three-layer system architecture, i.e., “sensing and perception” layer, “motion planner” layer, and “vehicle controller” layer [1,2].

Of the three layers, motion planner is responsible for generating a feasible reference trajectory for the low-level controllers to follow [3]. In the simple traffic environment with little or even no surrounding vehicles, this kind of motion planner has shown its effectiveness and has been successfully applied for autonomous driving [4]. However, when more complex environments with dense traffic are considered, the increased requirements of driving smoothness, comfort, and personalized adaptation complicate the motion planner and make it difficult to find a feasible reference trajectory within the time limit [1]. It has been found that experienced human drivers seem to work well in such complex environments without using a sophisticated algorithm to compute the optimal trajectory [5,6]. Moreover, human-like driving can improve the acceptance of autonomous vehicles by considering human personalities [7]. Therefore, how to learn and extract the behavior of human drivers and use the learned behavior to improve the motion planner has attracted increasing attention in the autonomous driving community [1,6,7,8]. To this end, during the last decades, some studies have been done to model human driving behavior and develop human-like control systems for vehicles. 

In the work carried out by [9], a fuzzy system is developed to model the behavior of an experienced human driver for parking a vehicle. A number of rules for the parking task can be extracted from the human experience and used to control the vehicle autonomously. Hybrid systems composed of both continuous models and discrete models are considered in [10] to model the driving behavior of drivers. A widely used hybrid model named autoregressive exogenous model (ARX) is selected because of its simplicity and high accuracy. In their model, human driving behavior is classified into different modes, and each mode corresponds to a continuous ARX model. By combining this kind of hybrid model with an MPC (model predictive control) controller, the driving behavior can be converted to the control commands for the vehicle [11]. 

Although the aforementioned rule- and model-based methods can reproduce human driving behavior in some cases, prior knowledge about the rule base and the model structure is usually required. For complex and dynamic scenarios, this kind of knowledge cannot be obtained easily in advance. In addition, human behaviors are strongly nonlinear, and it is very difficult to model human behaviors precisely using a physical model. Under such circumstances, learning-based methods that can learn directly from driving scenes and human demonstrators without prior knowledge are proposed. This kind of method is usually named learning from demonstration (LFD), which has been widely used in the humanoid robot domain to generate human-like movements [12]. For the vehicle control problem, the LFD method can help to reproduce the motion trajectories of the vehicle observed from human demonstrators [13].

Artificial neural networks (ANN) (both shallow and deep neural networks) [2,6,14,15,16,17,18] and Gaussian mixture models (GMM) [1,19,20,21] are commonly used methods for learning from driving scenes and drivers. Usually, driving data collected from drivers are used to train the ANN and the GMM offline, and, after training, the learned models can be connected with vehicle controllers to realize human-like control. Although these models have been applied to deal with complex traffic environments, the lack of online learning ability makes it difficult for these models to do the personalized adaptation during the learning process. Because of the online learning capability, reinforcement learning (RL) has been considered as a promising technology to improve the autonomous driving systems in recent years [22]. For instance, the Monte Carlo RL method is used by a collaborative driving system presented in [23] to improve the longitudinal control of vehicles. To solve both the longitudinal and the lateral control problems, a cooperative adaptive cruise control (CACC) system using the policy-gradient RL is built in [24]. For helping with the autonomous overtaking control, Q-learning (a kind of RL algorithm) is adopted by [25,26] to build the learning-based control systems. An actor-critic RL method is developed in [27] to improve the tracking precision and the driving smoothness for an autonomous vehicle. Although these RL-based systems can improve navigation and motion control for vehicles, the problem of learning personalized driving behavior and realizing the human-like control is not tackled by these studies.

This paper aims to develop a personalized behavior learning system (PBLS) based on RL with particular focus on the longitudinal speed control (LSC) problem for autonomous driving. The main contributions of this paper are as follows:

1. A reinforcement-learning-based system is proposed in this paper to learn the driver behavior and realize the human-like control. Based on RL, the system dynamics are not required and can be learned directly from the interaction between drivers and the driving environment.
2. By incorporating the controller into the learning system, the learned driving behavior can be converted to control commands for autonomous vehicles online, which realizes the personalized adaption for newly-involved drivers.

The remainder of this paper is organized as follows. Section 2 describes the system architecture of PBLS and gives the definition of different modules in the architecture. Then, Section 3 presents a solution algorithm for training PBLS. After that, two tests based on the driving simulator and the real driving data are shown in Section 4 and Section 5 to evaluate the performance of PBLS. Finally, Section 6 concludes the paper and gives future directions of the research.

## 2. Proposed Personalized Behavior Learning System

**Figure 1:**
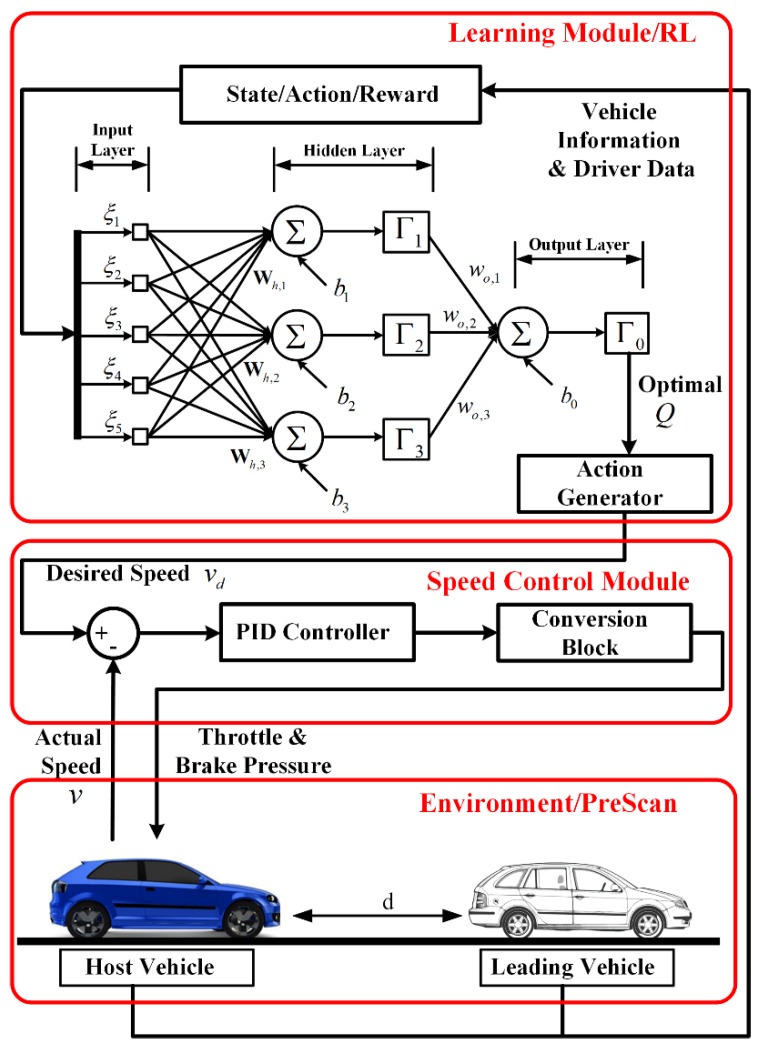
Architecture of the proposed personalized behavior learning system (PBLS). PID: proportion integration differentiation.

The system architecture for PBLS is shown in Figure 1, where the learning module is combined with a proportion integration differentiation (PID) controller to interact with the traffic environment during the learning process. In this study, the focus is on a typical LSC problem for car following. Considering the difficulties and the risks of testing the online learning system in real-world scenarios, the testing traffic scenarios are built in PreScan, a simulation tool for simulating vehicle dynamics and traffic environments [28]. The testing scenario for LSC consists of one host vehicle and one leading vehicle. The objective of the host vehicle is to follow the leading vehicle and try to keep a stable distance to the leading vehicle. Here, the host vehicle can either be controlled by a human driver or the proposed system. The driving data collected from the on-board sensors can be directly transferred to the learning module to activate the learning process. 

The learning module is based on RL and can learn the desired longitudinal speed from human drivers when they are controlling the host vehicle. Using a PID controller, the desired speed can be converted to the low-level control commands for throttle and brake pressure. In this way, the human car-following behavior can be reproduced.

### 2.1. Formulation of the Learning Module

The objective of the learning module is to learn the desired speed of the human driver, i.e., to track the speed trajectory of a human driver. Thus, the learning problem can be defined as a trajectory tracking problem for the given system $\dot{x}=f(x,u)$ and a desired trajectory $\left(x_{h},u_{h}\right)$. Let $s=x-x_{h}$, $a=u-u_{h}$, thus the trajectory tracking problem can be solved by a linear quadratic regulator (LQR) for minimizing the following cost function:

(1) $$J=\sum_{k=0}^{\infty }\left(s_{k}^{T}Cs_{k}+a_{k}^{T}Da_{k}\right),$$

such that,

(2) $$s_{k+1}=As_{k}+Ba_{k},$$

where k is the time index, $s_{k}$ is the state vector of the trajectory tracking problem, $a_{k}$ is the control action, A and B are matrices related to the system dynamics, and C and D are positive-definite matrices for weighting the cost function. The system dynamics is required for solving this problem based on the traditional LQR. For the real applications, the system dynamics are usually difficult to know in advance. In this case, an RL method can be used to learn the optimal solution to the trajectory tracking problem defined above.

Following [29], the cost at each time step can be defined by:

(3) $$r_{k}=s_{k}^{T}Cs_{k}+a_{k}^{T}Da_{k}.$$

It should be noted that $r_{k}$ is different from its counterpart in the traditional RL problem where $r_{k}$ is the reward at each time step and is used to formulate a maximization problem. Here, $r_{k}$ is related to the tracking error for the human behavior and thus should be minimized. For each state-action pair $\left(s_{k},a_{k}\right)$, the *Q* function can be defined following the Bellman equation and is given as follows:

(4) $$Q\left(s_{k},a_{k}\right)=\left[\begin{array}{cc} s_{k}^{T} & a_{k}^{T} \end{array}\right]\left[\begin{array}{cc} H_{ss} & H_{sa}\\ H_{as} & H_{aa} \end{array}\right]\left[\begin{array}{c} s_{k}\\ a_{k} \end{array}\right],$$

where $H_{ss}$, $H_{sa}$, $H_{as}$, and $H_{aa}$ are matrices related to the system dynamics and the weights of the cost function. By setting the derivative of Q with respect to $a_{k}$ to 0, i.e., $\nabla_{a_{k}}Q\left(s_{k},a_{k}\right)=0$, the optimal action can be derived and expressed by:

(5) $$a_{k}=-{\left(H_{aa}\right)}^{-1}H_{as}s_{k}=Ls_{k}.$$

For the car-following scenario considered here, the system dynamics are highly related to the distance between the host vehicle and the leading vehicle (denoted by d) as well as the speed of the host vehicle (denoted by v). Thus, for the system $\dot{x}=f(x,u)$, the following variables can be defined:

(6) $$x={\left[v\;d\right]}^{T},\;u=a.$$

Therefore, the state and the control action of the tracking problem can be defined as:

(7) $$s_{k}^{}={\left[\begin{array}{cc} s_{k,1} & s_{k,2} \end{array}\right]}^{T}={\left[\begin{array}{cc} v_{k}-v_{h,k} & d_{k}-d_{h,k} \end{array}\right]}^{T},\;a_{k}^{}=a_{k}-a_{h,k}$$

where $v_{k}$ and $v_{h,k}$ are the speeds of the host vehicle controlled by the learning system and the human driver at time step k, respectively, $d_{k}$ and $d_{h,k}$ are the distances controlled by the learning system and the human driver at time step k, respectively, and $a_{k}$ and $a_{h,k}$ are the accelerations of the host vehicle controlled by the learning system and the human driver at time step k, respectively.

Given the definition of state and action, the weights for the cost function can be determined as:

(8) $$C=\left[\begin{array}{cc} C_{1} & 0\\ 0 & C_{2} \end{array}\right],\;D=D,$$

where $C_{1}+C_{2}+D=1$. 

### 2.2. Function Approximation Using ANN

It can be seen from (4) that, to get the explicit *Q* value for each state-action pair, the system dynamics are required, i.e., the exact values of $H_{ss}$, $H_{sa}$, $H_{as}$, and $H_{aa}$ should be given. In the learning problem considered in this study, the system dynamics are not known in advance, thus an alternative way is used to learn the *Q* values from data samples and estimate $H_{ss}$, $H_{sa}$, $H_{as}$, and $H_{aa}$ for the purpose of calculating the optimal action. 

Assume that the *Q* function can be approximated by a linear function shown below:

(9) $$Q\left(s_{k},a_{k}\right)=Q\left(\xi_{k}\right)=\theta^{T}\xi_{k},$$

where

(10) $$\xi_{k}={\left[\xi_{k,1}\;\xi_{k,2}\;\xi_{k,3}\;\xi_{k,4}\;\xi_{k,5}\right]}^{T}={\left[\begin{array}{ccccc} s_{k,1}^{2} & s_{k,2}^{2} & 2s_{k,1}a_{k} & 2s_{k,2}a_{k} & a_{k}{}^{2} \end{array}\right]}^{T},$$

(11) $$\theta ={\left[\begin{array}{ccccc} \theta_{1} & \theta_{2} & \theta_{3} & \theta_{4} & \theta_{5} \end{array}\right]}^{T}.$$

Under the definition of Equation (9), Equation (4) can be rewritten as a linear function by setting:

(12) $$H_{ss}=\left[\begin{array}{cc} h_{1} & 0\\ 0 & h_{2} \end{array}\right],\;H_{as}=\left[\begin{array}{cc} h_{3} & h_{4} \end{array}\right],\;H_{sa}={\left[\begin{array}{cc} h_{5} & h_{6} \end{array}\right]}^{T},\;H_{aa}=h_{7}.$$

By substituting Equation (12) into Equation (4), the following equation can be obtained:

(13) $$Q\left(s_{k},a_{k}\right)=h_{1}s_{k,1}^{2}+h_{2}s_{k,2}^{2}+(h_{3}+h_{5})s_{k,1}a_{k}+(h_{4}+h_{6})s_{k,2}a_{k}+h_{7}a_{k}^{2}.$$

According to Equation (13), one can easily get:

(14) $$\theta_{1}=h_{1},\;\theta_{2}=h_{2},\;\theta_{3}=\left(h_{3}+h_{5}\right)/2,\;\theta_{4}=\left(h_{4}+h_{6}\right)/2,\;\theta_{5}=h_{7}.$$

In this way, $H_{ss}$, $H_{sa}$, $H_{as}$, and $H_{aa}$ can be constructed when θ is obtained.

From the definition of state and action, it can be seen that both of these two variables are continuous, thus traditional RL methods such as the standard Q-learning that can only deal with the discrete state and action space cannot be used here. Under such circumstances, the neural Q-learning (NQL) algorithm is adopted by this study to deal with the continuous problem. Under the framework of NQL, the continuous *Q* function can be approximated by an artificial neural network, and thus all the possible state and action values can be coped with.

As shown in Figure 1, a three-layer feed forward ANN similar to [29] is designed for the learning module. To guarantee the performance of the ANN, all the input variables should be normalized [30]. For the feed forward ANN considered in this study, the state and action defined in Equation (7) are normalized as follows.

(15) $$s_{k}^{}={\left[\begin{array}{cc} s_{k,1} & s_{k,2} \end{array}\right]}^{T}={\left[\begin{array}{cc} \frac{2\left(\Delta v_{k}-\Delta v_{\min}\right)}{\Delta v_{\max}-\Delta v_{\min}}-1 & \frac{2\left(\Delta d_{k}-\Delta d_{\min}\right)}{\Delta d_{\max}-\Delta d_{\min}}-1 \end{array}\right]}^{T},$$

where $\Delta v_{k}=v_{k}-v_{h,k}$, $\Delta d_{k}=d_{k}-d_{h,k}$, $\Delta v_{\max}$, and $\Delta d_{\max}$ are the maximum values of these two variables, and $\Delta v_{\min}$ and $\Delta d_{\min}$ are the minimum values of these two variables. Here, $\Delta v_{\min}$, $\Delta v_{\max}$, $\Delta d_{\min}$, and $\Delta d_{\max}$ can be obtained from the data. In this way, both $s_{k,1}$ and $s_{k,2}$ can be normalized into a range between −1 and 1.

Similarly, the action can be normalized as:

(16) $$a_{k}^{}=\frac{2\left(\Delta a_{k}-\Delta a_{\min}\right)}{\Delta a_{\max}-\Delta a_{\min}}-1.$$

In this way, all the elements of $\xi_{k}$ are normalized into [−1, 1]. Let $\xi_{k}$ be the input vector for the input layer, then the feed forward ANN can be defined by its activation functions $\Gamma_{i},\;i=0,1,2,3$ for each node. The output of ANN is the optimal *Q* function, which can be expressed by:

(17) $$Q\left(\xi_{k}\right)=\Gamma_{0}\left(\sum_{i=1}^{3}\left(w_{o,i}\Gamma_{i}\left(W_{h,i}\xi_{k}+b_{i}\right)\right)+b_{0}\right),$$

where $W_{h,i}$ ($W_{h,i}=[w_{h,i}^{1}\cdots w_{h,i}^{5}]$ for five input variables) is the weight vector for the ith node in the hidden layer, $w_{o,i}$ is the weight for the link from the ith hidden node to the output node, $b_{j}$ is the bias for the ith hidden node, and $b_{0}$ is the bias of the output node. When the optimal *Q* value is obtained, the elements of the parameter vector θ can be calculated by:

(18) $$\theta_{l}\left(\xi_{k}\right)=\frac{\partial Q\left(\xi_{k}\right)}{\partial \xi_{k,l}},\;l=1,2,3,4,5.$$

Then, the optimal action can be derived from Equation (5) through reconstructing $H_{aa}$ and $H_{as}$ from θ according to Equation (14).

### 2.3. Speed Control Module

Given the action, the desired speed can be easily derived from Equation (16) and calculated by:

(19) $$a_{k}=\frac{\Delta a_{\max}(a_{k}+1)-\Delta a_{\min}(a_{k}-1)}{2}+a_{h,k},v_{d,k+1}=v_{d,k}+a_{k}\Delta t,$$

where $v_{d,k}$ and $v_{d,k+1}$ are the desired speeds for the kth and the k+1th time step, respectively. 

The speed control module can then convert the desired speed to control commands for the throttle and the break pressure control of the host vehicle using a PID controller [31].

(20) $$y\left(t\right)=K_{p}\left[v_{e}\left(t\right)+\frac{1}{T_{I}}\int_{0}^{t}v_{e}\left(\tau \right)d\tau +T_{D}\frac{dv_{e}\left(t\right)}{dt}\right],$$

where $v_{e}\left(t\right)$ is the tracking error between the desired speed and the actual speed, $K_{p}$ is the proportional gain, $T_{I}$ is the integral time, $T_{D}$ is the derivative time, and y(t) is the output of the controller, which can be converted to the throttle and the breaking control commands by a conversion block. Both the PID controller and the conversion block are embedded in PreScan and implemented as a module named “Path follower”. In this study, the default parameter values (provided by PreScan) for the PID controller are applied for all of the experiments. These default parameters provided by PreScan are set as: $K_{p}=20$, $K_{p}/T_{I}=0.3$, and $K_{p}/T_{D}=3.0625$.

## 3. Training Algorithm for PBLS

Technically, the goal of the learning system is to find the optimal *Q* value and its corresponding parameter vector θ. Temporal difference (TD) learning [32] is a method to solve this problem by making the TD error defined by Equation (21) approach zero:

(21) $$e_{k}=r_{k}+Q\left(s_{k+1},a_{k+1}\right)-Q\left(s_{k},a_{k}\right).$$

Here, the feed forward ANN is used to accomplish this goal. For N time steps, the errors should be cumulated to formulate the loss function for ANN. For ease of calculation, a quadratic loss function is defined as follows:

(22) $$E=\underset{\mathrm{cumulative}\;\mathrm{error}}{\underset{︸}{\frac{1}{2N}\sum_{k=1}^{N}e_{k}{}^{2}}}+\underset{\mathrm{regularisation}\;\mathrm{term}}{\underset{︸}{\frac{\lambda }{2}\left(\sum_{i=1}^{3}{\left(w_{o,i}\right)}^{2}+\sum_{j=1}^{3}\sum_{l=1}^{5}{\left(w_{h,j}^{l}\right)}^{2}\right)}}.$$

The first term of Equation (22) is related to the sum of squares of errors, which should be minimized by ANN. The second part of Equation (22) is named weight decay term, which is used here to avoid over-fitting by reducing the magnitude of the weights [33].

From the definition of $e_{k}$, it can be seen that the bias of the hidden node does not affect the loss function and thus can be removed from Equation (17). Let $\Gamma_{0}$ be a linear function and $\Gamma_{i},\;i=1,2,3$ be a number of hyperbolic tangent functions, then Equation (17) can be rewritten as:

(23) $$Q\left(\xi_{k}\right)=\sum_{i=1}^{3}\left(w_{o,i}\tanh\left(W_{h,i}\xi_{k}+b_{i}\right)\right).$$

The hyperbolic tangent function is selected, as it is a typical activation function for ANN and has been proven to be effective in many practical cases [30].

Thus, according to Equations (18) and (21), the elements of θ can be obtained from:

(24) $$\begin{array}{ll} \theta_{l}\left(\xi_{k}\right) & =\sum_{i=1}^{3}\left(w_{o,i}w_{h,i}^{l}\left(1-\tanh^{2}\left(W_{h,i}\xi_{k}+b_{i}\right)\right)\right)\\ & =\sum_{i=1}^{3}\left(w_{o,i}w_{h,i}^{l}\right)-\sum_{i=1}^{3}\left(w_{o,i}w_{h,i}^{l}\tanh^{2}\left(W_{h,i}\xi_{k}+b_{i}\right)\right)\\ & \approx \sum_{i=1}^{3}\left(w_{o,i}w_{h,i}^{l}\right). \end{array}$$

The second part $\sum_{i=1}^{3}\left(w_{o,i}w_{h,i}^{l}\tanh^{2}(W_{h,i}\xi_{k}+b_{i})\right)$ of Equation (24) is very small when the weights and the biases are small and can be ignored, as suggested by [29]. Therefore, θ is only related to the weight matrix of the ANN and can be calculated when $w_{o,i}$ and $w_{h,j}^{l}$ are updated. As the objective of ANN is to minimize the loss function shown in Equation (22), the gradient decent method can be used here to update the weights. The weights for the output layer can be updated by:

(25) $$\begin{array}{ll} w_{o,i,u} & =w_{o,i,u-1}-\alpha {\left(\frac{\partial E}{\partial w_{o,i}}\right)}_{u-1}\\ & =w_{o,i,u-1}-\alpha \left(\frac{1}{N}{\sum_{k=1}^{N}\left(\frac{\partial e_{k}}{\partial w_{o,i}}\right)}_{u-1}+\lambda w_{o,i,u-1}\right), \end{array}$$

where u is the updating index, and the network is updated every N time steps. Similarly, the weights for the hidden layer and the biases can be calculated by:

(26) $$w_{h,i,u}^{l}=w_{h,i,u-1}^{l}-\alpha \left(\frac{1}{N}\sum_{k=1}^{N}{\left(\frac{\partial e_{k}}{\partial w_{h,i}^{l}}\right)}_{u-1}+\lambda w_{h,i,u-1}^{l}\right)$$

and

(27) $$b_{i,u}=b_{i,u-1}-\alpha \frac{1}{N}\sum_{k=1}^{N}{\left(\frac{\partial e_{k}}{\partial b_{i}}\right)}_{u-1}.$$

The key issue right now is how to get the gradients of weights and biases at each updating step, i.e., the terms $\partial e_{k}/\partial w_{o,i}$, $\partial e_{k}/\partial w_{h,i}^{l}$, and $\partial e_{k}/\partial b_{i}$. To this end, the back propagation (BP) algorithm can be used to train the ANN via a mini-batch training method. Frequently updating the weights of the neural network, e.g., step-by-step update with N = 1, may lead to poor generalization and unstable learning curves, especially for learning unstable human behaviors. To overcome this limitation, the network weights are usually updated every N steps (N > 1) by using a small batch of data. This kind of training method is named mini-batch training and has been widely used for training neural networks [34]. In this paper, the mini-bath training is used to train the feedforward ANN, and, as suggested by [34], a small N with the value 10 (between two and 32) is selected. This kind of setting can help to avoid the bias of newly collected driving data and guarantee a relatively smooth learning curve in our experiment. Based on BP, the whole algorithm for the learning system is developed and shown in Algorithm 1.

**Algorithm 1:** Pseudo-code for PBLS**Initialization**(1)Initialize ANN in terms of α, $w_{o,i}$, $W_{h,i}$ and $b_{i}$(2)Initialize *Q* function Q=0, state $s_{0}$ and action $a_{0}$**Action generation**(3)**For** each time step $k<N_{k}$ **do**Observe the state $s_{k}$ at the current step and get the recorded state $s_{k-1}$ and action $a_{k-1}$.Get the reward $r_{k-1}$ through Equation (3)Get the action $a_{k}$ through Equation (5)Get the desired speed $v_{d,k}$ through Equation (19)Get the control commands though Equation (20)Calculate ANN-related parametersForward propagation, get Q through Equation (23)Back propagation, get:The error for the output $\delta_{o}\leftarrow e_{k}$ through Equation (21)The error for the hidden layer:$z_{i}=W_{h,i}\xi +b_{i},\;\delta_{h,i}^{}\leftarrow \left(w_{o,i}\delta_{o}\right)\Gamma_{i}^{\prime }\left(z_{i}\right),\;i=1,\;2,\;3$Calculate the gradients for i=1, 2, 3 and l=1, 2, 3, 4, 5:$\partial e_{k}/\partial w_{o,i}\leftarrow \Gamma_{i}\delta_{o}$$\partial e_{k}/\partial w_{h,i}^{l}\leftarrow \xi_{l}\delta_{h,i}$$\partial e_{k}/\partial b_{i}\leftarrow \delta_{h,i}$ Let$\Delta w_{o,i}\leftarrow \Delta w_{o,i}+\partial e_{k}/\partial w_{o,i}$$\Delta w_{h,i}^{l}\leftarrow \Delta w_{h,i}^{l}+\partial e_{k}/\partial w_{h,i}^{l}$$\Delta b_{i}\leftarrow \Delta b_{i}+\partial e_{k}/\partial b_{i}$**Mini-batch updating**g.**IF** Mod($N_{k}/N=0$) (N=10) for i=1, 2, 3 and l=1, 2, 3, 4, 5 **do**$w_{o,i}\leftarrow w_{o,i}-\alpha \left[\frac{1}{N}\Delta w_{o,i}+\lambda \Delta w_{o,i}\right]$$w_{h,i}^{l}\leftarrow w_{h,i}^{l}-\alpha \left[\frac{1}{N}\Delta w_{h,i}^{l}+\lambda w_{h,i}^{l}\right]$$b_{i}\leftarrow b_{i}-\alpha \frac{1}{N}\Delta b_{i}$(4)**End if**(5)**End for**ANN: artificial neural network.

## 4. Experiments with Constant Speed

The proposed learning system (PBLS) is tested in a simulation platform built by PreScan and Matlab/Simulink in this section. As mentioned in Section 2, the vehicle information and the driver data are required by the learning system. In PreScan, both the host and the leading vehicles are equipped with a virtual lidar system, a Global Positioning System (GPS), and vehicle-to-vehicle (V2V) communication systems. The vehicle information in terms of location, speed, and distance between the host vehicle and the leading vehicle can be obtained through these on-board systems. 

**Figure 2:**
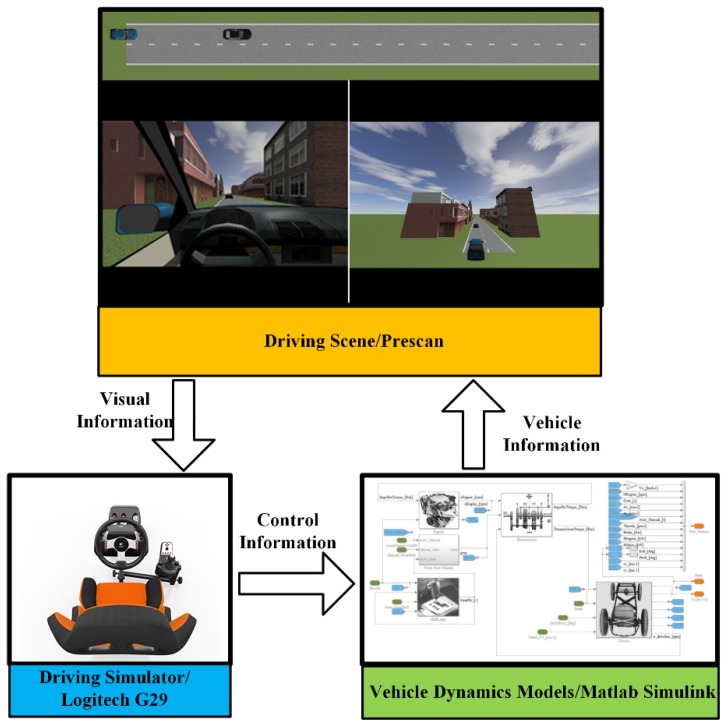
Driving simulator used for the experiment.

As shown in Figure 2, driver data can be collected by the Logitech G29 driving simulator through the human-in-the-loop experiments. For real applications, the driving data can be obtained through the on-board sensing system. The vehicles involved in the experiments are modeled by the typical 2-D vehicle dynamics models (single-track model), which are embedded in Matlab/Simulink. The traffic environment and the driving scene are simulated in PreScan, which is connected to the driving simulator and provides drivers with the visual information. Two groups of experiments with different speed profiles—constant speed (CS) and variant speed (VS)—for the leading vehicle are carried out to evaluate the performance of the proposed system. 

In all the tests, the weight values are set as $C_{1}=C_{2}=D=1/3$ to guarantee that each part of the cost $r_{k}$ has the same importance. Other parameters for PBLS are shown in Table 1, which are chosen according to experience and can guarantee a stable performance of PBLS.

### 4.1. Experimental Settings

**Figure 3:**
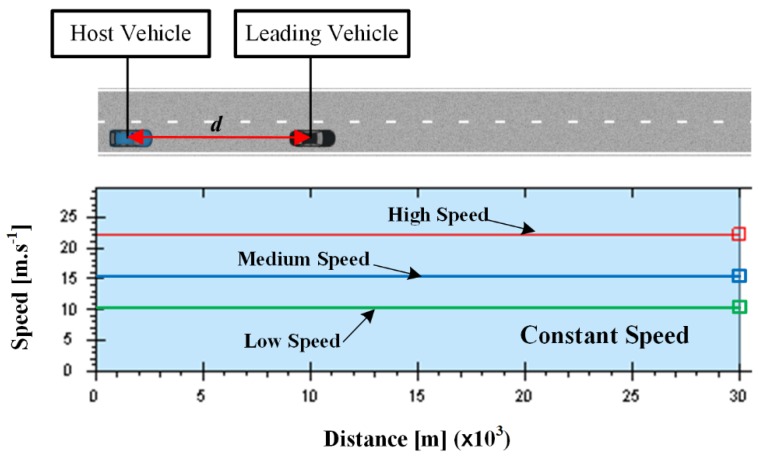
Driving scene for constant speed scenario.

The driving scene used by the constant speed scenarios is shown in Figure 3. A straight two-lane urban road with a length of 30 km is considered. In the test, the driver is asked to drive the host vehicle first, and then the driving data collected from the driver are transferred to PBLS, which is used to control the host vehicle in the same scene and learn the driving behavior from the collected driving data online. When the learning algorithm is converged, PBLS can reproduce the learned behavior by setting the learning rate as zero. 

In the first test, the leading vehicle keeps a constant speed, and three speed profiles, namely, low speed (L, 10 m·s^−1^), medium speed (M, 15 m·s^−1^), and high speed (H, 22 m·s^−1^), are designed to form three different test scenarios. To test the adaptive learning ability of the proposed system, two drivers (A and B) are involved and asked to follow the leading vehicle in all three scenarios. Then, the learning system is triggered to learn the driving behavior from these two drivers. It should be noted here that the focus of this study is to develop a personalized learning system that has the ability to adapt to different driving behaviors. This kind of adaptation can be tested by involving two different drivers in this section. Analytical work involving more drivers can be considered in our future study to analyze the algorithm performance under various kinds of driving behaviors.

RMSE (Root Mean Square Error) can be used to measure the learning error of the learning system, which is calculated by:

(28) $$\mathrm{RMSE}(z)=\sqrt{(1/N_{k})\sum_{k=0}^{N_{k}-1}{\left(z_{k}-{\hat{z}}_{k}\right)}^{2}}$$

where $z_{k}$ is the data point related to the learning system at step k, and ${\hat{z}}_{k}$ is the observed data from human drivers at step k.

### 4.2. Experimental Results

**Figure 4:**
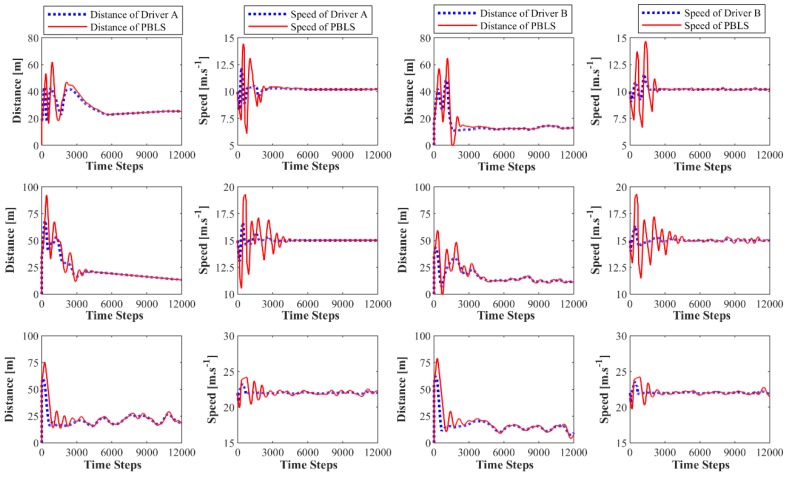
Learning curves for the distance and the speed of two drivers: the figures in the first row are for the low speed scenario; the figures in the second row are for the medium speed scenario; the figures in the third row are for the high-speed scenario.

**Figure 5:**
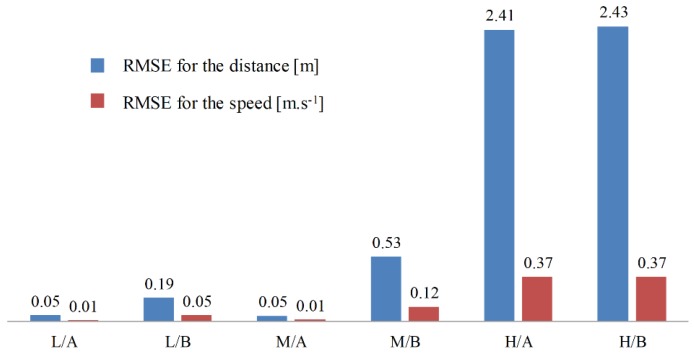
Root Mean Square Error (RMSE) for the constant speed scenarios.

Figure 4 presents the learning curves of PBLS for different speed scenarios. In all three scenarios, the learning system can learn the stable distance and the speed curves within 5000 time steps (250 s). As shown in Figure 5, in the low speed scenario, the learning RMSE for both the speed and the distance of two drivers can be kept at a very low level close to zero. However, with the growth of speed for the leading vehicle, the performance of PBLS gets worse with RMSE for the speed increasing from 0.01 m·s^−1^ to 0.37 m·s^−1^ and RMSE for the distance increasing from 0.05 m to 2.43 m. This result is reasonable, as in the low-speed scenario, both drivers can perform well in keeping a stable distance to the leading vehicle. In this situation, the curves for speed and distance are very smooth without large fluctuation after around 5000 time steps, and thus PBLS performs better in this scenario.

In all three scenarios, PBLS shows a better performance on reproducing the behavior of Driver A than Driver B with lower RMSE for Driver A. This is mainly because Driver A has more experience in driving and can keep a relatively stable curve for both speed and distance.

## 5. Experiments with Variant Speed

In the previous section, the learning ability of the proposed system was tested in scenarios with constant speed. In this section, three driving scenes with variant speeds for the leading vehicle are considered. In the first two driving scenes, the whole test is similar to the constant speed case, except that a traditional adaptive cruise control (ACC) system is considered here to make a comparison with PBLS, which is the focus of this section. In the third driving scene, driving data collected from real vehicles on the real road are used to test the learning system. The driver (Driver A) with more driving experience is involved in this section. In the following test, PBLS only learns from Driver A.

The ACC system is a widely used longitudinal speed control system, which is designed to assist drivers to keep a pre-set time headway between the host vehicle and the leading vehicle [35]. The time headway is defined as the ratio of the distance (d) to the speed of the host vehicle (v). The desired time headway for ACC is set as 1.8 s in the test, which can keep d between 20 m and 40 m when the leading vehicle has a speed between 10 m·s^−1^ and 20 m·s^−1^. In this way, the d kept by ACC and PBLS can be ranged to the same level, which helps to make a fair comparison.

Two indicators suggested by [36] are used here to evaluate and compare the performance of PBLS and ACC on driving comfort and smoothness. These two indicators are given by:

(29) $$J_{1}=\frac{a_{mean}}{v_{mean}},$$

(30) $$J_{2}=\frac{da}{dt},$$

where the driving comfort is measured by $J_{1}$, which is obtained by dividing the average acceleration $a_{mean}$ by the average speed $v_{mean}$, and the driving smoothness is measured by $J_{2}$, which is the jerk of the vehicle. 

The driving comfort is considered low when $J_{1}$ is at a high level, while a high driving smoothness corresponds to a low and stable $J_{2}$.

### 5.1. Driving Scene I

**Figure 6:**
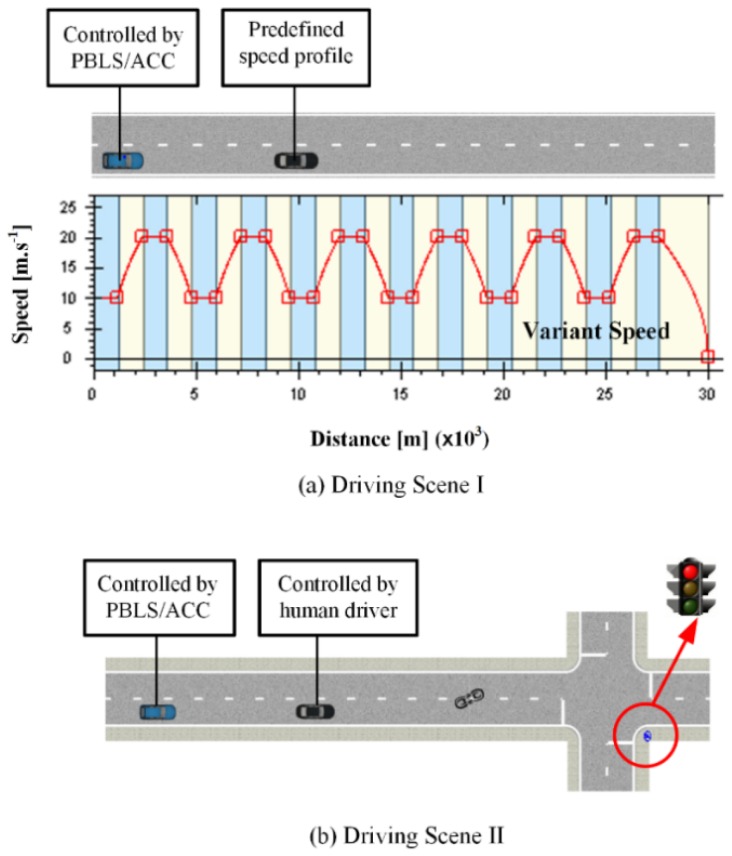
Driving scenes for variant speed scenarios.

As shown in Figure 6a, in the first driving scene, the road layout is the same as in Section 4, while the speed of the leading vehicle changes between 10 m·s^−1^ and 20 m·s^−1^ during the whole test. For data collection, the driver in the host vehicle is asked to follow the leading vehicle with variant speed in the first run. After that, the proposed PBLS is triggered for behavior learning. In this case, the algorithm runs for 80,000 time steps (around 1 h for convergence) for learning and then runs for 40,000 time steps by setting the learning rate as zero to reproduce the learned behavior.

**Figure 7:**
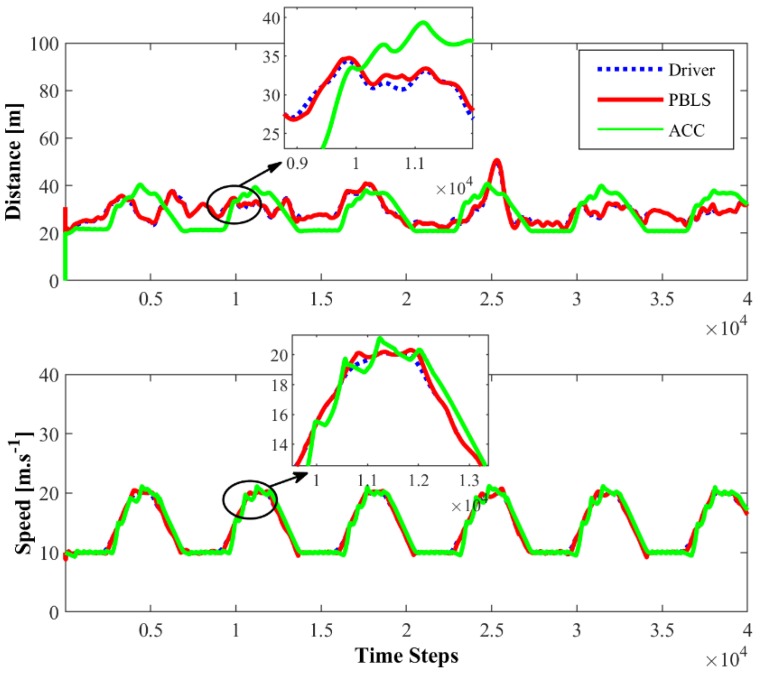
Distance and speed for Driving Scene I.

Figure 7 presents the distance and the speed comparison among the driver with PBLS and ACC in the first driving scene. PBLS performs well in learning from the driver with the distance and the speed curves close to the driver, which means the learning error (RMSE) of PBLS is at a very low level.

**Figure 8:**
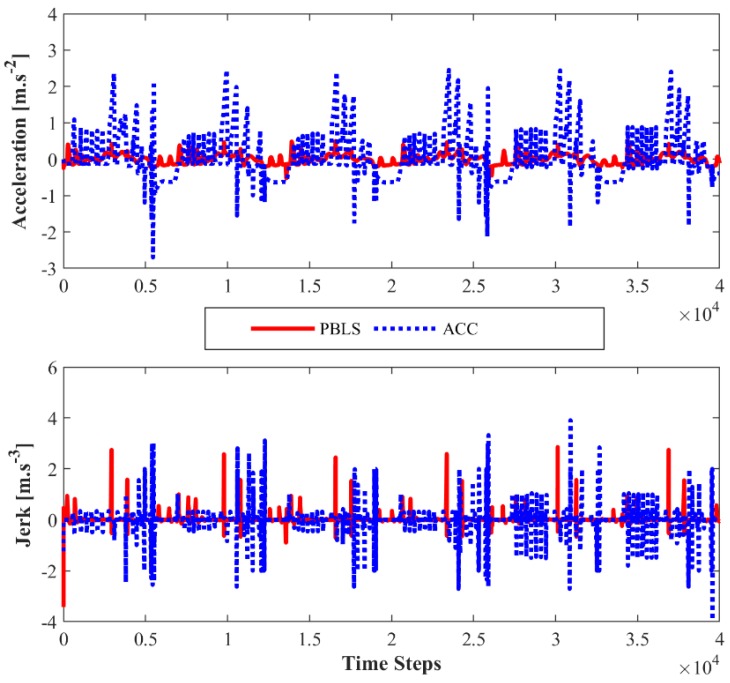
Acceleration and jerk (*J*_2_) for Driving Scene I.

Compared to PBLS, the speed of ACC fluctuates more greatly, especially when the speed is close to 20 m·s^−1^. As shown in Figure 8, the acceleration and the jerk ($J_{2}$) of ACC vary significantly during the whole test, while PBLS can keep a relatively stable curve for both the acceleration and the jerk. Thus, PBLS can provide better driving smoothness than ACC. 

### 5.2. Driving Scene II

In the second driving scene presented in Figure 6b, the leading vehicle is controlled by a human driver without predefined speed profiles. Therefore, in the data collection phase, both the host vehicle and the leading vehicle are driven by human drivers. A typical intersection with a traffic light is involved to form Driving Scene II.

In this scene, the leading vehicle is asked to go through the intersection according to the traffic light, and the host vehicle follows the leading vehicle all the time. The traffic light changes following the order: yellow, red, and green. The time for the yellow light is set as 5 s (100 steps), and the red light lasts for 40 s (800 steps). There is no time limit for the green light, which guarantees that both vehicles can pass through the intersection.

**Figure 9:**
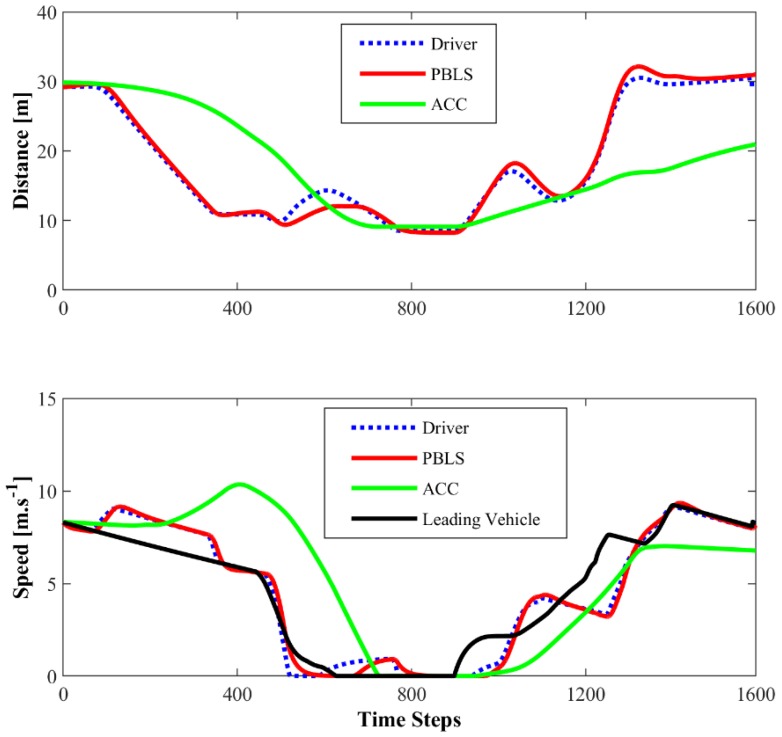
Distance and speed for Driving Scene II.

The initial speed for the leading vehicle and the host vehicle is 8 m·s^−1^, and the initial distance between these two vehicles is 30 m. It can be seen from Figure 9 that, because of the yellow and the red light, the leading vehicle slows down in the first 600 steps (30 s) when it is approaching the stop line. Then, it restarts and speeds up after 300-step waiting at the stop line.

**Figure 10:**
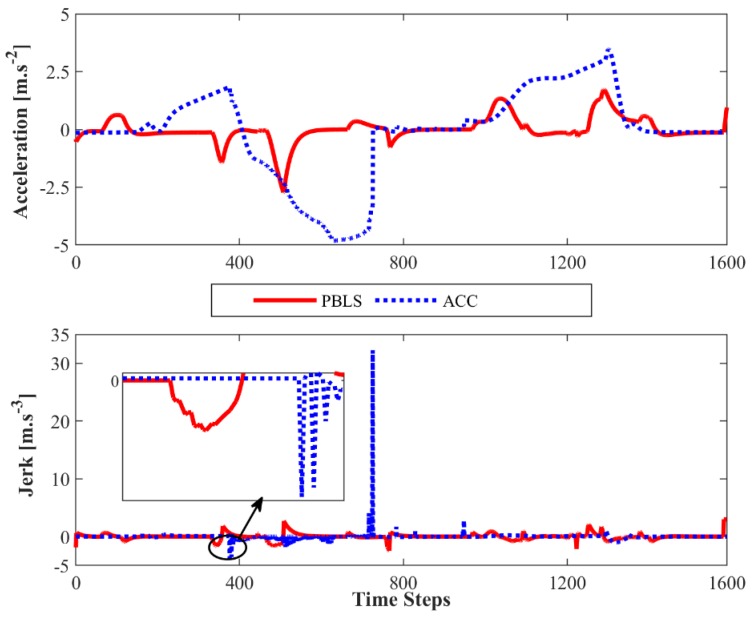
Acceleration and jerk (*J*_2_) for Driving Scene II.

**Figure 11:**
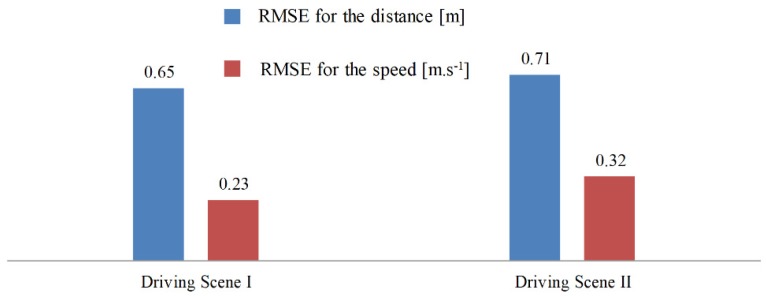
RMSE in different scenes.

**Figure 12:**
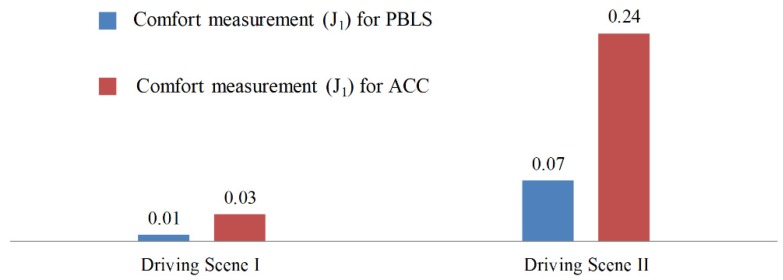
Comfort measurement (*J*_1_) for different systems in different scenes.

In this test, the algorithm runs for 12,000 time steps (600 s) to get convergence, which means the whole test needs to repeat 10 times. Similar to the test in Driving Scene I, after learning, the learning rate of the algorithm is set as zero to reproduce the learned behavior. As shown in Figure 10, compared with ACC, PBLS has better driving smoothness with smoother acceleration and jerk trajectories. From Figure 11, it can be seen that PBLS can reproduce the behavior of the driver who controls the host vehicle with a very low RMSE, while the difference between the curves of ACC and the driver is very large (see Figure 9). Thus, compared with ACC, PBLS is more consistent with the driver’s behavior and habits. Except for the driving smoothness, PBLS also performs better than ACC in the driving comfort. As shown in Figure 12, the $J_{1}$ of PBLS is much smaller than the $J_{1}$ of ACC.

### 5.3. Driving Scene III

**Figure 13:**
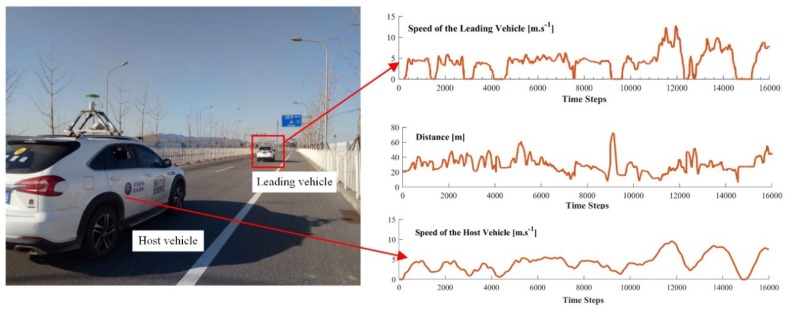
An illustration of the real driving data and vehicles.

In the third driving scene, as shown in Figure 13, two real vehicles are involved for collecting the real driving data. The Beijing Institute of Technology (BIT) intelligent vehicle [37] is used as the host vehicle in this work. This vehicle is equipped with on-board sensing systems to capture the speed and the distance information. The detailed description of the host vehicle can be found in [37]. Both host and leading vehicles are driven by human drivers. The driver in the leading vehicle is asked to drive along a straight road with a changeable speed. 

**Figure 14:**
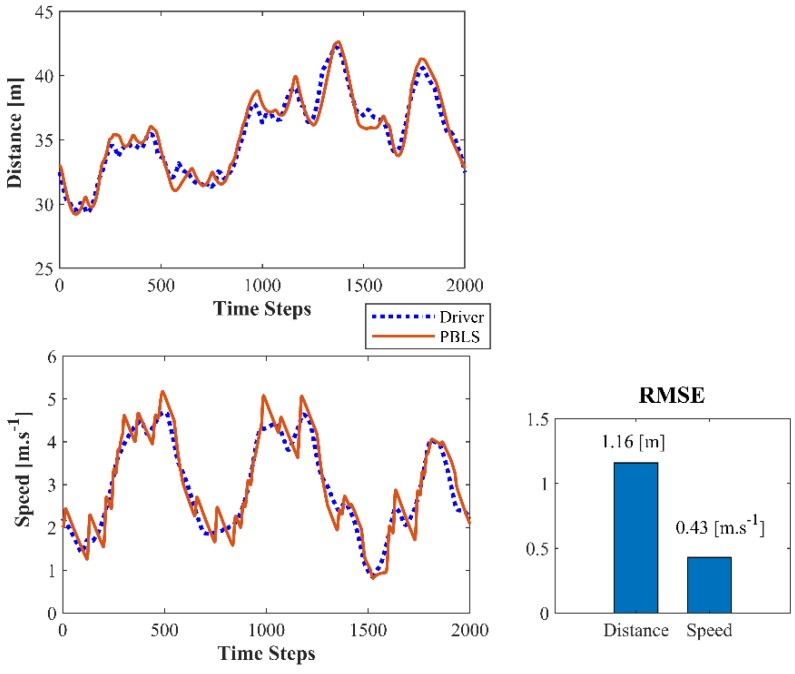
Test result for Driving Scene III.

After the data collection process, real driving data are used to test the learning system. Testing the on-line learning and control system directly on a real-world road is highly risky, as slight learning deviations may lead to severe safety issues for both testing and surrounding vehicles. Thus, in this study, the real driving data are used to reproduce the observed real driving scene in PreScan, where the simulated leading vehicle follows the speed profile observed from the real world. The real behavior data collected from the host vehicle are used to train the PBLS in PreScan. The collected data shown in Figure 13 are divided into eight groups, and each group contains the data collected from 2000 time steps. Seven groups of data are used to train the algorithm, and the remaining group is used for testing. The test result is shown in Figure 14. 

Compared with Driving Scenes I and II, PBLS in Driving Scene III performs slightly worse with higher RMSE for both distance and speed. This is mainly because the real driving data are noisier than the simulation data, especially when the leading vehicle has a changeable speed.

## 6. Conclusions

A personalized behavior learning system (PBLS) was proposed in this paper to learn the human driving behavior from demonstrations. PBLS is based on a reinforcement learning method named neural *Q*-learning (NQL), which can approximate the *Q* function in a continuous state and action space, such that the human-like longitudinal speed control (LSC) problem can be solved properly. To train PBLS online, a batch-updating algorithm based on back-propagation (BP) was developed. 

A series of driving simulator experiments with different speed profiles for the leading vehicle were carried out to evaluate the performance of PBLS. In all the experiments, PBLS kept a low learning error, especially for the driver who had a stable operation. In the test with variant speed, by learning from an experienced driver, PBLS achieved higher driving comfort and smoothness than the traditional adaptive cruise control (ACC) system. 

As mentioned in Section 4, this study focused on developing a personalized behavior learning system that can adapt to different drivers. In future work, a systematic analysis involving more drivers will be conducted to investigate the effects of different drivers and driving styles on the performance of the learning system. 

## Figures and Tables

**Table 1:** Parameters for PBLS.

Scenarios	α	λ	$\Delta v_{\max}\;[m\cdot s^{-1}]$	$\Delta v_{\min}\;[m\cdot s^{-1}]$	$\Delta d_{\max}\;[m]$	$\Delta d_{\min}\;[m]$	$\Delta a_{\max}\;[m\cdot s^{-2}]$	$\Delta a_{\min}\;[m\cdot s^{-2}]$
CS/10 [m·s^−1^]	0.1	0.0005	15	−15	80	0	4	−4
CS/15 [m·s^−1^]	0.1	0.0005	20	−20	80	0	4	−4
CS/22 [m·s^−1^]	0.1	0.0005	25	−25	80	0	6	−6
VS/Scene I	0.01	0.05	25	−25	80	0	4	−4
VS/Scene II	0.01	0.5	25	−25	80	0	8	−8

CS: constant speed; VS: variant speed.
